# Liver Resection and Transplantation Following Yttrium-90 Radioembolization for Primary Malignant Liver Tumors: A 15-Year Single-Center Experience

**DOI:** 10.3390/cancers15030733

**Published:** 2023-01-25

**Authors:** Daniel Aliseda, Pablo Martí-Cruchaga, Gabriel Zozaya, Macarena Rodríguez-Fraile, José I. Bilbao, Alberto Benito-Boillos, Antonio Martínez De La Cuesta, Luis Lopez-Olaondo, Francisco Hidalgo, Mariano Ponz-Sarvisé, Ana Chopitea, Javier Rodríguez, Mercedes Iñarrairaegui, José Ignacio Herrero, Fernando Pardo, Bruno Sangro, Fernando Rotellar

**Affiliations:** 1 HPB and Liver Transplant Unit, Department of General Surgery, Clinica Universidad de Navarra, University of Navarra, 31008 Pamplona, Spain; 2 Institute of Health Research of Navarra (IdisNA), 31008 Pamplona, Spain; 3 Nuclear Medicine Department, Clinica Universidad de Navarra, University of Navarra, 31008 Pamplona, Spain; 4 Interventional Radiology, Department of Radiology, Clinica Universidad de Navarra, University of Navarra, 31008 Pamplona, Spain; 5 Anesthesiology Unit, Clinica Universidad de Navarra, University of Navarra, 31008 Pamplona, Spain; 6 Department of Oncology, Clinica Universidad de Navarra, University of Navarra, 31008 Pamplona, Spain; 7 Liver Unit and HPB Oncology Area, Clinica Universidad de Navarra and CIBEREHD, 31008 Pamplona, Spain

**Keywords:** radioembolization, liver transplantation, liver resection, hepatocellular carcinoma, intrahepatic cholangiocarcinoma

## Abstract

**Simple Summary:**

Radioembolization is a locoregional therapy used in primary liver malignancies with different applications depending on the treatment goal. The aim of this retrospective study was to evaluate postoperative and long-term survival outcomes of patients with unresectable or high biological risk HCC and ICC treated with RE that were finally rescued to liver surgery with curative intent. In a cohort of 34 patients, we assessed that liver resection and transplantation after RE seem safe and feasible with adequate short-term outcomes. Moreover, long-term outcomes after RE and LR were optimal, with a 10-year OS rate greater than 50% for HCC and ICC patients. On the other hand, the 10-year OS rates from RE were also greater than 50% for patients with HCC downstaged or bridged to LT.

**Abstract:**

Radioembolization (RE) may help local control and achieve tumor reduction while hypertrophies healthy liver and provides a test of time. For liver transplant (LT) candidates, it may attain downstaging for initially non-candidates and bridging during the waitlist. Methods: Patients diagnosed with HCC and ICC treated by RE with further liver resection (LR) or LT between 2005–2020 were included. All patients selected were discarded for the upfront surgical approach for not accomplishing oncological or surgical safety criteria after a multidisciplinary team assessment. Data for clinicopathological details, postoperative, and survival outcomes were retrospectively reviewed from a prospectively maintained database. Results: A total of 34 patients underwent surgery following RE (21 LR and 13 LT). Clavien–Dindo grade III-IV complications and mortality rates were 19.0% and 9.5% for LR and 7.7% and 0% for LT, respectively. After RE, for HCC and ICC patients in the LR group, 10-year OS rates were 57% and 60%, and 10-year DFS rates were 43.1% and 60%, respectively. For HCC patients in the LT group, 10-year OS and DFS rates from RE were 51.3% and 43.3%, respectively. Conclusion: Liver resection after RE is safe and feasible with optimal short-term outcomes. Patients diagnosed with unresectable or high biological risk HCC or ICC, treated with RE, and rescued by LR may achieve optimal global and DFS rates. On the other hand, bridging or downstaging strategies to LT with RE in HCC patients show adequate recurrence rates as well as long-term survival.

## 1. Introduction

Liver cancer is the fourth most commonly diagnosed cancer and the third leading cause of cancer-related death worldwide [1]. Hepatocellular carcinoma (HCC) and intrahepatic cholangiocarcinoma (ICC) account for 80% and 15% of primary liver cancers, respectively [2]. Unfortunately, most cases are detected at advanced stages, and potentially curative treatments cannot be applied as the first option [3]. A wide range of treatments, including locoregional therapies (LRTs) (transarterial chemoembolization (TACE), stereotactic body radiation therapy, intensity modulated radiation therapy, or radioembolization) and systemic drugs, are used for patients with unresectable primary liver tumors [4,5]. Most LRTs are focused on delaying progression by eliminating or reducing tumor burden [6]. Radioembolization (RE) delivers radiation by injecting 90Y-loaded microspheres through the hepatic arteries, thus eliminating tumor cells [7]. In fact, RE arrests tumor growth in more than 90% of the treated patients [8], achieving disease control rates between 75% and 100% [9,10]. Our preliminary experience [11], then further substantiated by other research groups, has shown that RE produces clinically significant reductions in tumor size (downsizing) in patients who were not initially candidates for LT or LR [12]. Evidence also supports RE as a bridge to LT and for HCC downstaging before transplantation [11,13], resulting in longer time-to-progression than other LRTs [14]. On the other hand, the capacity to induce contralateral hypertrophy when applied as a lobar treatment—called radiation lobectomy—may allow LR with curative intent in patients initially considered unresectable not only due to tumor features but also due to insufficient future liver remnant (FLR) [15]. Consequently, LR and LT after RE may provide survival benefits to some selected patients not deemed suitable for surgical treatment at diagnosis. Over the years, post-RE surgical experience has increased, but data is scarce and heterogeneous in regard to morbidity, mortality, and survival [16].

The aim of this study was to analyze postoperative and survival outcomes of LR and LT after RE in a cohort of patients diagnosed with HCC and ICC that were deemed unresectable and/or unsuitable for LT at diagnosis. 

## 2. Materials and Methods

A single-center, retrospective study was performed. This study was conducted according to the recommendations of the Declaration of Helsinki, and the protocol was approved by the Institutional Review Board (ref. 2021.056). Informed consent was obtained from patients before surgery and any RE procedure. 

### 2.1. Patient Evaluation and Eligibility 

#### 2.1.1. Inclusion Criteria

Adult patients ≥ 18 y.o. who were diagnosed with primary liver tumors (HCC or ICC) and underwent LR or LT after RE between December 2005 and December 2020 at our center were included in this study. Patients were evaluated by our institutional HepatoPancreatoBiliary Oncology (MDT), and an indication for RE was decided.

#### 2.1.2. Criteria for Considering Patients Unsuitable for LR or LT 

Exclusion criteria for LR in HCC were based on the decision of the MDT, taking into account a combination of parameters: a minimum >30% FLR (>40% in cirrhotic patients) and indocyanine green retention (after 2013) tests as per reported by Makuuchi et al. were used for patient selection [17]. Regarding ICC, in healthy liver, exclusion criteria for LR were only if vascular infiltration of main trunks was observed, leading to an unlikely R0 resection. In cirrhosis, similar criteria as displayed above were used for ICC. Patients outside the Pamplona criteria from 1991 to 2012 [18,19] and outside Up-to-7 (for deceased donors) or Up-to-8 (for living donors) from 2012 onwards were considered non-candidates for LT [20].

#### 2.1.3. Indications for Yttrium-90 Radioembolization

It should be noted that the indication for RE has evolved over time. Therefore, there was not a uniform protocol for Yttrium-90 radioembolization with strict fixed criteria. Initially, patients with HCC or ICC were treated with purely palliative intent. Some patients with an unresectable disease treated with palliative intention eventually became resectable/transplantable [11]. As experience increased, the indication migrated from cases treated with palliative intent to a downstaging intention. Posteriorly, we identified the capacity of RE to induce FLR hypertrophy [21,22]. Accordingly, a pre-surgical intention was introduced, with the perspective of inducing tumor response and contralateral hypertrophy (in patients with insufficient FLR). Furthermore, the achievement of these RE-induced effects is not immediate. Considering that these tumors often have aggressive biological behavior, RE also acts as a test of time, allowing us to more accurately select those patients who will benefit from radical surgical treatment.

In patients who would benefit from LT, downstaging was attempted in order to reduce tumor burden within the applicable criteria at every moment. Bridging was considered as the use of RE to prevent disease progression while the patient was on the waiting list. 

### 2.2. Yttrium-90 Radioembolization

#### 2.2.1. Eligibility 

Indication for RE treatment and aim was assessed for each patient by the HepatoPancreatoBiliary Oncology MDT. Our eligibility criteria have been detailed elsewhere [23]. Briefly, patients were only considered for RE if they had an Eastern Cooperative Oncology Group (ECOG) performance status of 0 or 1, as well as preserved liver (absence of ascites and serum total bilirubin <2 mg/dL), hematological (platelet count >40/pL), and renal function (serum creatinine <2 mg/dL), no contraindication to angiography, and were able to provide informed consent. Additionally, the following conditions were considered exclusion criteria:

(a) A lung shunt fraction >20% or an estimated dose of radiation to the lungs > 30 Gy;
(b) Previous stereotactic body radiation therapy to the liver;
(c) Presence of collateral vessels feeding extrahepatic organs that cannot be corrected by angiographic techniques.

#### 2.2.2. Radioembolization Protocol

Patients were treated following our previously reported RE protocol [23]. Those submitted to RE underwent an angiographic mapping of the abdominal and hepatic arteries. Planar scans of the lung and liver area in anterior and posterior views were acquired after injection of 99mTc-labelled albumin macroaggregated albumin (^99m^Tc-MAA) into selected arterial branches followed by SPECT (until 2006) and SPECT/CT scans (from 2006). RE was delivered using Yttrium-90 resin microspheres (SIR-Spheres; Sirtex Medical Limited, North Sydney, NSW, Australia). The method for calculating the prescribed 90Y activity evolved over time. Initially, the BSA method was used in all cases. Subsequently, after a retrospective assessment carried out in our center [23], a modified BSA method was used for whole liver treatments and the partition model for lobar or lobar-extended RE. Currently, personalized dosimetry considering optimal absorbed doses by tumoral and non-tumoral volumes is used. SIR-Spheres were injected within 15 days of the ^99m^Tc-MAA scan. In all cases, a same-day calibration 3 GBq vial was used (44  ±  2.6 million spheres per vial) [24]. RE-related toxicities were classified according to the National Cancer Institute Common Terminology Criteria for Adverse Events v5 [25] and the assessment of RE-induced liver disease [26]. 

### 2.3. Response to Radioembolization, Re-Evaluation, and Eligibility for Liver Resection or Transplantation

Computed tomography or MRI scans and laboratory tests were performed over intervals of 1 to 3 months after RE. Serial measurements of tumor-specific biomarkers (alpha-fetoprotein for HCC and carbohydrate antigen 19.9 for ICC) were also conducted. For patients initially not suitable for LT and after adequate downstaging, patients were listed if they fulfilled the current LT criteria, and priority was based on the Model for End-Stage Liver Disease (MELD) score. For patients with tumors initially considered unresectable that were downstaged with RE, LR with curative intent was indicated based on the individual benefit that patients could derive from radical surgical treatment and always ensuring sufficient volume and function of the FLR. In patients with initial insufficient FLR, patients were considered candidates for surgery if there was no tumor progression and the FLR reached sufficient volume and function adjusted to the planned surgery and patient/liver conditions. In combination with the above-described situations, in patients with biologically aggressive tumors, the time taken to achieve RE objective(s) was also used as a test of time tool to assess the response and evolution of the disease in order to better select those patients who could benefit from radical surgical treatment.

### 2.4. Surgical Outcomes

Liver resection was classified into three groups according to the extent: minor (one or two Couinaud’s segments), major (three to four Couinaud’s segments), and major extended (five or more Couinaud’s segments). Intraoperative parameters, including operative time, blood loss, red blood cell (RBC) transfusion, clamping time, and cold organ ischemia, were prospectively collected. Post-hepatectomy liver failure (PHLF), hemorrhage, and bile leakage were evaluated using the criteria of the International Group for the Study of Liver Surgery [27,28,29]. Pure laparoscopic liver resection (LLR) was defined as a surgery entirely performed through 12, 10, and 5 mm ports. The IWATE criteria [30] and the Southampton scoring system [31] were used to determine the difficulty level of the LLR and the risk of intraoperative events, respectively. Postoperative follow-up studies included CT or MRI scans, blood cell counts, serum biochemistry, and tumor-specific biomarkers 1, 6, and 12 months after the surgery and, subsequently, every year.

### 2.5. Data Collection 

The following data were collected from a prospectively-maintained database: demographics, characteristics of the disease (etiology, Child–Pugh status, BCLC stage), clinical assessments (ECOG performance status, encephalopathy), characteristics of RE, perioperative outcomes, hospital course, morbidity at 30 and 90 days (according to the Clavien–Dindo classification [32]), mortality, pathology (R1 resection rate, macro- and micro-vascular invasion, degree of necrosis), and long-term oncological data (status, recurrence/metastases, site of recurrence).

### 2.6. Statistical Analysis

Quantitative variables are presented as the mean (standard deviation) or median (range). Qualitative variables are presented as numbers and percentages. The Student’s *t*-test was used for inter-group comparisons for normally distributed continuous variables; otherwise, the Mann–Whitney U test was used. The Chi-square or Fisher’s exact test was applied to compare non-normally distributed categorical variables. Overall survival (OS) was calculated as the time (months) from the date of RE and liver surgery (LR or LT) to death or last follow-up date; disease-free survival (DFS) was calculated as the time (months) from the date of RE and liver surgery until the date of tumor recurrence or metastases. Kaplan–Meier curves were displayed to evaluate long-term OS and DFS. A *p*-value less than 0.05 was considered statistically significant. Statistical analyses were conducted using STATA version 16 (StataCorp, College Station, Texas, 77845, USA).

## 3. Results

During the study period, 257 consecutive patients diagnosed with HCC (*n* = 219) or ICC (*n* = 38) were treated with RE at our center. Among them, 26 HCC patients (11.9%) and eight ICC patients (21.1%) underwent LR or LT after RE, respectively. The mean patient age of the subgroup of patients who underwent surgery was 64.7 y.o (±1.2), and 32 patients were male (94.1%). The whole cohort’s and HCC patient’s baseline characteristics are shown in Table 1 and Table 2, respectively. 

### 3.1. Radioembolization Characteristics and Outcomes

Twenty-nine patients (85.3%) received a single RE treatment before surgery, four patients (11.8%) received two sessions, and one patient (2.9%) received three RE treatments. The median 90Y activity was 1.2 GBq (range 0.3–3.25 GBq), with higher activity in the LR group (1.5 Gbq) compared with the LT group (0.5 Gbq). Absorbed doses by tumor obtained with the partition model were collected, and the median absorbed doses by the tumor of the resection and transplantation groups were 116 Gy (92–204) and 91 Gy (35–150), respectively. According to National Cancer Institute Common Terminology Criteria for Adverse Events v5.0 [25], three patients (14.2%) presented a grade III adverse event due to RE treatment, and one patient (2.9%) suffered RE-induced liver disease [26]. No RE-related mortality was registered. 

### 3.2. Surgical Outcomes

#### 3.2.1. Liver Resection

Liver resection was performed in 21 patients. The median time from RE to resection was 8 months (2–32). Nine patients (42.9%) underwent pure LLR and 12 open hepatectomies. Regarding the extent of the hepatectomy, there were 3 minor (14.3%), 13 major (61.9%), and 5 major extended (23.8%) hepatectomies. Major resections were distributed as follows: nine right hepatectomies (42.9%; 3 required partial diaphragmatic resection due to severe RE-related liver adhesions), one left hepatectomy (4.8%), three central hepatectomies (14.3%), three right trisectionectomies (14.3%) and two left-extended hepatectomies (9.5%; 1 extended to the caudate lobe and the other extended to the ventral segment VIII).

The median operative time was 328 minutes (135–803). Seven patients (33.3%) required intraoperative RBC transfusion, 6 of which were open liver resections. Among ICC patients, associated lymphadenectomy of the hepatic pedicle, hepatic artery, and celiac trunk was performed. Four patients (19.0%) required major vascular reconstruction, including the right supra hepatic vein, the portal vein (including the portal bifurcation in one case), and the inferior vena cava. 

#### 3.2.2. Laparoscopic Liver Resection

Nine patients underwent LLR with no conversion to open or assisted surgery. Eight hepatectomies (88.9%) were major or major-extended LLR; part of this experience has been previously reported [33]. The IWATE difficulty score [30] was low, advanced, and expert in two, two, and five cases, respectively. The median risk score for adverse intraoperative events was nine (4–10), implying high risk. Median clamping time and blood loss in this subgroup were 82.5 minutes (35–107) and 325 cc (50–1000), respectively. 

#### 3.2.3. Liver Transplantation

Thirteen HCC patients underwent LT. The median time from RE to transplantation was 10 months (7–14). Six patients (46.2%) were initially outside LT criteria and received RE after downstaging. One patient (7.7%) underwent living donor transplantation, and 12 received deceased donor organs: 11 donations after brain death and one after cardiac death. LT was performed using the piggyback technique in all patients. The median operative time was 322 minutes (251–387). Ten patients (76.9%) received an intraoperative transfusion of fresh frozen plasma (median of three units (2–5)). Intraoperative transfusion of RBC was performed in seven patients (53.8%; median of two units (1–4)), and platelet transfusion in five patients (38.5%; median of one unit (1–2)). The mean cold ischemia time was 5 h and 42 min (±35 min). 

### 3.3. Postoperative Outcomes

#### 3.3.1. Liver Resection

The median hospital stay was 5 days (3–17). Four patients (19.0%) presented Clavien–Dindo grade III-IV complications. One required re-operation due to inadvertent small bowel perforation. Biliary leakage was registered in four patients: one treated conservatively and three requiring US-guided or transparietohepatic drainage (during postoperative hospital stay). At the 90-day follow-up, two patients died (9.5%). Both were treated by right trisectionectomy with vascular reconstruction. In addition, one of them had received whole-liver RE at the beginning of our experience. In both cases, the cause of death was a multiorgan failure, including one grade C PHLF. Surgical free margin (R0) was achieved in 18 patients (85.7%), and positive microscopic margins (<1 mm) were registered in three patients (14.3%). No positive macroscopic margins were found. No viable tumor (pT0) was reported in one patient (4.8%). Necrosis between 50–99% and <50% was observed in 12 and 8 patients, respectively.

#### 3.3.2. Liver Transplantation

The median hospital stay was 6 days (5–16). One Clavien–Dindo grade III-IV complication was registered due to active bleeding on the hepatic artery anastomosis that required re-operation. There were no biliary complications. Two patients (15.4%) required readmission because of persistent fever (>38 °C; one adverse drug reaction and one gastroenteritis). There were no deaths within 90 days. Liver resection and LT postoperative outcomes are shown in Table 3.

### 3.4. Survival Analysis

#### 3.4.1. Liver Resection

*Hepatocellular Carcinoma*

**Figure 1:**
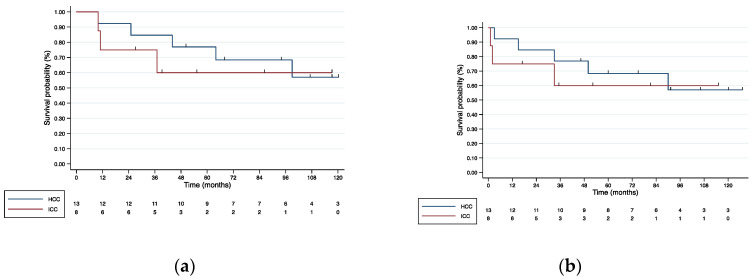
(**a**) Kaplan–Meier OS curves from RE for resected patients; (**b**) Kaplan–Meier OS curves from liver resection.

After a median follow-up of 94 months (10–176), 6 recurrences and 5 deaths occurred (four due to disease progression and one because of cardiac-associated comorbidities). From RE, 3-, 5- and 10-year OS rate was 84.6%, 76.9%, and 57.0%, respectively (Figure 1a). From LR, 3-, 5- and 10-year OS rate was 76.9%, 68.4%, and 57.0%, respectively (Figure 1b). From RE, 3-, 5- and 10-year DFS was 61.5%, 53.9%, and 43.1%, respectively (Figure S1a). Recurrence sites are shown in Table S1.

*Intrahepatic Cholangiocarcinoma*

After a median follow-up of 38 months (10–120), one patient presented recurrence localized in the contralateral hemiliver, and three patients died (one of them free of disease). From RE, 3-, 5- and 10-year OS and DFS were 75%, 60%, and 60%, respectively (Figure 1a and Figure S1a, Supplementary Materials). From LR, 1-, 3-, and 5-year OS was 75%, 60%, and 60%, respectively (Figure 1b). 

#### 3.4.2. Liver Transplantation

**Figure 2:**
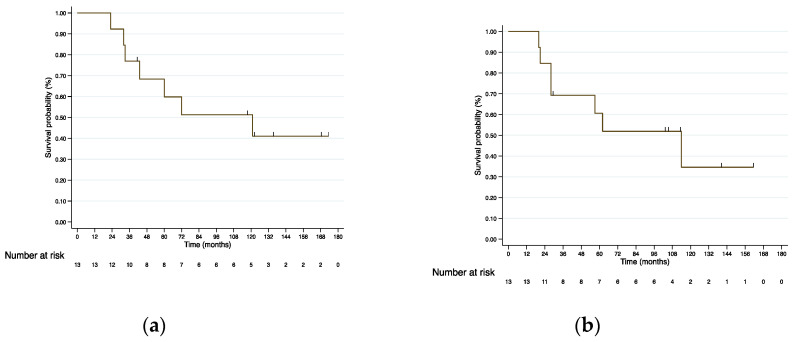
(**a**) Kaplan–Meier OS curves from RE for LT patients; (**b**) Kaplan–Meier OS curves from LT.

After a median follow-up of 72 months (23–173), there were three recurrences and seven deaths (two due to disease progression and five due to associated comorbidities). From RE, 3-, 5- and 10-year OS was 76.9%, 68.4%, and 51.3%, respectively (Figure 2a). From LT, 3-, 5-, and 10-year OS was 69.2%, 60.1%, and 34.6%, respectively (Figure 2b). From RE, 3-, 5-, and 10-year DFS was 69.2%, 60.6%, and 43.3%, respectively (Figure S1b)

## 4. Discussion

Over the years, it has been shown that RE can lead to tumor downstaging and downsizing, be used as a bridge to transplantation, and—when administered regionally—cause atrophy and compensatory hypertrophy of the contralateral liver, which are great advantages in LR and liver transplantation [11,15]. As previously demonstrated by our group, these characteristics allow some patients initially diagnosed with unresectable lesions to become candidates for curative surgery [11]. Recently, the LEGACY study showed a 3-year survival rate of 86.6% in patients with unresectable solitary HCC (≤8 cm), using RE as a purely ablative treatment [9]. The same study demonstrated how the downstaging efficacy to surgery (resection or transplantation) further improves the survival outcomes, reaching a 3-year OS of 92.8% in the subgroup of patients rescued to surgery [9]. However, LR in patients with previous RE is complex, as in most patients, it combines major or extended resections, large tumors, and intense parenchymal fibrosis, which may hamper pedicle dissection and transection [33,34,35]. Furthermore, post-RE adhesions to surrounding organs (e.g., diaphragm or abdominal wall) can also increase the difficulty [33,36]. In 2009, Gulec et al. reported the first post-RE hepatectomy [37]. In subsequent years, studies on the safety and short-term results of post-RE resections were published, with variable and heterogeneous results [16,36].

### 4.1. Liver Resection

#### 4.1.1. Postoperative Outcomes

The registered postoperative results in our series are similar to those previous reports showing a median hospital stay of 3 to 9 days [16,38,39], major complication rates of 16% to 78%, and 90-day mortality between 0% and 33% [16]. On the other hand, our outcomes seem similar to those obtained for major hepatectomies in patients without previous RE [40,41]. Consequently, we can consider that LR after RE is feasible and safe. However, it is particularly relevant to highlight the importance of proper patient selection, and for this purpose, it is necessary to consider pre-surgical aspects (e.g., FLR, remnant function) as well as dosimetry and RE extension parameters. This was also concluded from the multicenter P4S study [36]. Beyond being deemed safe, it could be considered that LR after RE may achieve the same textbook outcomes in liver surgery (TOLS) as any other LR, as our initial experience in major LLR suggests [33]. On the other hand, the possibility of a laparoscopic approach in these patients is a breakthrough since LLR in patients with HCC has been shown to be beneficial in terms of postoperative outcomes compared with open liver resection [42,43]. In the presence of previous RE, our group was the first to prove the safety and successful results of major LLR with a median hospital stay of 2 days and a major complication and 90 day-mortality rates of 22.2% and 0%, respectively. Further, we show that these results comply 77.8% with the actual TOLS and are not worse than the outcomes obtained in patients who underwent major LLR without previous RE [33,44,45]. 

#### 4.1.2. HCC Survival Outcomes

To the best of our knowledge, there are no 5-year survival outcomes in the literature for patients who underwent LR post-RE, and the largest experience was published by Gabr et al. with a 3-year survival of 86% [38]. After RE, the present series reports a 3-, 5-, and 10-year OS of 84.6%, 76.9%, and 57.0%, respectively. These results are striking as they are similar to those reported in patients resected for early HCC with good liver function and within the Milan criteria [46]. Moreover, survival seems slightly superior in comparison with those seen in HCC patients with initially unresectable lesions who underwent treatment by ALPPS and post-PVE surgery [47]. In addition to the aforementioned features, RE adds the valuable “*test of time*” that allows assessment of disease progression and selection of patients that will benefit most from surgical treatment. However, this evidence should be taken with caution until confirmed by future larger and prospective series. Similarly, studies showing HCC recurrence post-RE LR are scarce. Two studies have shown recurrence rates of 29% and 68.8%, with median recurrence times of 34.3 and 13.8 months, respectively [38,48]. The results presented in our series are similar to those reported in the literature, with a recurrence rate of 46.2% and a 5-year DFS of 53.9%. This data is interesting, taking into account that 5-year DFS in patients who underwent surgery for early HCC within the Milan criteria and without previous RE varies between 21% and 57%, as published elsewhere [46].

#### 4.1.3. ICC Survival Outcomes

Five-year OS after upfront resection in ICC initially resectable patients varies between 20% and 40% [49]. In patients considered unresectable, chemotherapy is the most widely used therapeutic option, and the expected median OS is 11.7 months [50]. Recently, the combination of RE and chemotherapy aiming downstaging with surgical rescue has appeared as a promising option [51]. There are few published series describing survival after surgery and RE in ICC patients. Two studies showed 1- and 5-year OS of 60% and 56% after a median follow-up of 15.6 and 46 months, respectively [51,52]. These results seem consistent with those obtained in our series with a 5-year OS of 60% after LR. A recent prospective phase II clinical trial reported optimal results when combining chemotherapy and RE in ICC patients; the trial showed a decrease in lesion size, 22% downstaging, and 98% of control of the disease [51]. In that study, ICC recurrence, with a median follow-up of 46 months, was 22.2%, and the 2-year DFS rate was 66.8%. In the present series, after a median follow-up of 38 months, a recurrence rate of 12.5% and a 5-year DFS of 60% were registered, these being encouraging outcomes considering those reported in the literature.

### 4.2. Liver Transplantation

The role of LRTs prior to transplantation in patients diagnosed with HCC is currently under study. Some publications show improvements in OS and recurrence in favor of LRTs despite having less favorable baseline tumor characteristics [53]. RE is a well-established treatment and can be used prior to LT to achieve downstaging or bridging in patients outside the transplant criteria or awaiting LT [54]. Although most of the published experiences in downstaging or bridging are based on TACE [55], recently, Gabr et al. reported a 10-year OS and DFS of 60% and 43%, respectively, in HCC patients treated with RE followed by LT [56]. Consequently, this group favors RE over other locoregional therapies in patients diagnosed with HCC based on its capacity to achieve greater downstaging, extend the time until progression, and reduce waitlist dropout [54]. The results published by Gabr et al. [56] are outstanding and even similar to those obtained in LT for HCC within the Milan criteria [57]. Compared with our series, the difference in OS is notorious. This could probably be due to the fact that the downstaging of patients in our series was performed taking into account wider transplantation criteria (Pamplona criteria from 1990 to 2012 and subsequently Up-to-7). However, it is noteworthy that only two patients in this series died due to causes related to previous HCC or LT and that the recurrence outcomes and DFS are consistent with those published for other LT series after RE [56]

It is important to consider our results as the development of the knowledge, applicability, and indications of RE. This heterogeneous series shows how, in the beginning, patients treated with RE with palliative intent became resectable or reached LT, revealing the downstaging capacity [11]. Subsequently, RE was used for bridging, achieving a reduced rate of progression and stability of the disease [54]. Finally, the knowledge of dosimetry applied to both the tumor and the remaining healthy liver parenchyma has allowed it to be used to treat the tumor while achieving volume and functional hypertrophy of the FLR [58]. As a consequence of this evolution, RE is now an accepted treatment tool in the treatment of HCC and is considered a potentially curative therapy as well as a valuable pre-operative tool in cirrhotic and non-cirrhotic patients [9,15,21,59].

Limitations of this study include its retrospective nature, relatively limited number of patients, and a selection bias inherent to this patient population. As corresponds to the historical observation of a technique that has evolved over time, this series implies a considerable grade of heterogeneity in terms of indication, technique, or dosimetry of RE. At the same time, given the retrospective nature of a prospective surgical database, it has not been possible to perform an intention-to-treat analysis. For this reason, the results must be considered with caution. Moreover, in some patients with HCC, the survival achieved cannot be fully attributed to RE treatment because they previously received other therapies (as shown in Table 1). However, the fact that some patients received previous locoregional or systemic treatments shows the evolution of aggressive tumors that—in most patients—did not respond and progress to these previous treatments. This aspect reinforces the use of RE. On the other hand, the result herein presented also provides an enriching perspective and shows the real evolution of a promising technique that is being solidly established. With this in mind, further prospective cohorts and randomized studies are needed to explore RE as an alternative to other locoregional treatments and assess its downstaging capacity, its implication in surgery, and particularly how it affects survival and local recurrence outcomes.

## 5. Conclusions

Liver resection after RE is safe and feasible with adequate short-term outcomes. To achieve the best possible postoperative results, the correct selection of patients is paramount. Furthermore, patients diagnosed with unresectable HCC or ICC, treated with RE, and rescued by LR seem to achieve excellent global and DFS rates. On the other hand, bridging or downstaging strategies to LT with RE in patients diagnosed with HCC show adequate recurrence rates as well as long-term survival. 

## Figures and Tables

**Table 1:** Patient’s pre-operative characteristics.

	Whole Cohort (*n* = 34)	Resection (*n* = 21)	Transplantation (*n* = 13)
Age (years) ^a^	64.7 (1.2)	66.3 (1.1)	62 (1.3)
Sex (M:F)	32:2	19:2	13:0
Tumor type			
HCC	26 (76.4)	13 (61.9)	13 (100)
ICC	8 (23.6)	8 (38.1)	0 (0.0)
BMI (kg/m2) ^a^	28.1 (0.7)	27.4 (0.7)	29.1 (1.2)
ASA score ^b^	3 (2–4)	3 (2–4)	3 (3–4)
Comorbidities			
Hypertension	14 (41.2)	12 (57.1)	2 (15.4)
Cardiopathy	15 (44.1)	8 (38.1)	7 (53.8)
Diabetes	10 (29.4)	5 (23.8)	5 (38.5)
COPD	2 (5.9)	2 (9.5)	0 (0.0)
Chronic renal injury	4 (11.8)	3 (14.3)	1 (7.7)
Prior abdominal surgery	13 (38.2)	10 (29.4)	3 (23.1)
Pre-operative tumor size (cm) ^a^	6.3 (0.7)	8.2 (0.8)	3.4 (1.4)
Total bilirubin (mg/dl) ^b^	0.9 (0.4–6.8)	0.7 (0.4–1.7)	1.7 (0.5–6.8)
Prior liver procedure	8 (23.6)	4 (19.0)	4 (30.7)
Resection	0 (0.0)	0 (0.0)	0 (0.0)
Ablation	4 (11.8)	2 (9.5)	2 (15.4)
Portal vein embolization	2 (5.9)	2 (9.5)	0 (0.0)
TACE	2 (5.9)	0 (0.0)	2 (15.4)
RE treatment			
Whole liver radiation	3 (8.9)	3 (14.3)	0 (0.0)
Lobar extended	5 (14.7)	4 (19.0)	1 (7.7)
Lobar	13 (38.2)	6 (28.6)	7 (53.8)
Segmental	13 (38.2)	8 (38.1)	5 (38.5)
Pre-RE systemic treatment ^+^	10 (29.4)	10 (47.6)	0 (0.0)
Post-RE systemic treatment	8 (23.5)	8 (38.1)	0 (0.0)

Data are expressed as *n* (%) unless otherwise specified. ^a^ Values are mean (standard deviation) or ^b^ median (range). ^+^ Including sorafenib or immunotherapy. BMI, body mass index; ASA, American Society of Anesthesiologists; HCC, hepatocellular carcinoma; ICC, intrahepatic cholangiocarcinoma; COPD, chronic obstructive pulmonary disease; RE, radioembolization; TACE, transarterial chemoembolization.

**Table 2:** Pre-operative characteristics of HCC patients.

	HCC Cohort (*n* = 26)	Resection (*n* = 13)	Transplantation (*n* = 13)
Cirrhosis (Child–Pugh)	23 (88.5)	10 (76.9)	13 (100)
Grade A	20 (87.0)	9 (90.0)	11 (84.6)
Grade B	3 (13.0)	1 (10.0)	2 (15.4)
Grade C	0 (0.0)	0 (0.0)	0 (0.0)
Staging BCLC			
BCLC A	9 (34.6)	2 (15.4)	7 (53.8)
BCLC B	14 (53.8)	8 (61.5)	6 (46.2)
BCLC C	3 (11.5)	3 (23.1)	0 (0.0)
Etiology of HCC			
Alcoholic cirrhosis	9 (34.6)	1 (7.7)	8 (61.5)
HCV	8 (30.8)	6 (46.2)	2 (15.4)
HBV	2 (7.7)	0 (0.0)	2 (15.4)
NASH	1 (3.8)	1 (7.7)	0 (0.0)
Hemochromatosis	2 (7.7)	2 (15.4)	0 (0.0)
Cryptogenic	4 (15.4)	3 (23.1)	1 (7.7)
Serum AFP (ng/mL) ^a^	5.99 (1.7–10659)	2685 (1.8–10659)	9.75 (1.7–805)

Data are expressed as *n* (%) unless otherwise specified. ^a^ median (range). BCLC, Barcelona clinic liver cancer; HCV, hepatitis C virus; HCC, hepatocellular carcinoma; HBV, hepatitis B virus; NASH, nonalcoholic steatohepatitis; AFP, alpha-fetoprotein.

**Table 3:** Postoperative outcomes.

	Whole Cohort (*n* = 34)	Resected (*n* = 21)			Transplanted (*n* = 13)
Postoperative Outcomes		Whole	Open (*n* = 12)	LLR (*n* = 9)	
Hospital stay (day) ^a^	6 (3–17)	5 (3–17)	9.5 (4–17)	3.5 (3–11)	6 (5–16)
Overall complication	13 (38.2)	9 (42.9)	6 (50.0)	3 (33.3)	4 (30.8)
Clavien–Dindo III-IV	5 (14.7)	4 (19.0)	2 (16.7)	2 (22.2)	1 (7.7)
PHLF (ISGLS)					
PHLF C	1 (2.9)	1 (4.8)	1 (4.8)	0 (0.0)	0 (0.0)
PHH (ISGLS)					
PHH C	1 (2.9)	0 (0.0)	0 (0.0)	0 (0.0)	1 (7.7)
Bile leakage (≥grade B) (ISGLS)	3 (8.8)	3 (14.3)	2 (16.7)	1 (11.1)	0 (0.0)
Re-operation	2 (5.9)	1 (4.8)	0 (0.0)	1 (11.1)	1 (7.7)
90 days readmission	4 (11.8)	2 (9.5)	1 (4.8)	1 (11.1)	2 (15.4)
90 days mortality	2 (5.9)	2 (9.5)	2 (16.7)	0 (0.0)	0 (0.0)

Data are expressed as *n* (%) unless otherwise specified. ^a^ median (range). PHLF, posthepatectomy liver failure; PHH, posthepatectomy hemorrhage; ISGLS, Study Group of Liver Surgery.

## Data Availability

The data presented in this study are available upon request from the corresponding author.

## Supplementary Materials

The following supporting information can be downloaded at: https://www.mdpi.com/article/10.3390/cancers15030733/s1, Table S1: Recurrence outcomes; Figure S1: (**a**) Kaplan–Meier DFS curves from diagnosis for LR group according to diagnosis and (**b**) Kaplan–Meier DFS curves from liver transplantation
