# Supplementary material for: Liver Resection and Transplantation Following Yttrium-90 Radioembolization for Primary Malignant Liver Tumors: A 15-Year Single-Center Experience
Source: Cancers (Basel). 2023 Jan 25;15(3):733. doi: 10.3390/cancers15030733 (PMC9913597; doi:10.3390/cancers15030733)
Supplement: Supplementary file 1 [file cancers-15-00733-s001.zip › cancers-2143126-supplementary.pdf]

Supplementary Materials

Tables

Table S1. Recurrence outcomes.

	Resected (n= 21)		Transplanted (n=13)
**Number of recurrences**	**7 (33.3)**		**3 (23.1)**
**Tumor type**			
• HCC	6 (46.2)		3 (23.1)
• ICC	1 (12.5)		-
Site of first recurrence	**ICC**	**HCC**	HCC
**Liver only**	**1 (12.5)**	**3 (14.3)**	**1 (7.7)**
**Liver + nonliver sites**	**0 (0.0)**	**1 (4.8)**	**1 (7.7)**
• Liver + bone	0 (0.0)	0 (0.0)	1 (7.7)
• Liver + lung	0 (0.0)	1 (4.8)	0 (0.0)
**Extrahepatic**	**0 (0.0)**	**2 (9.5)**	**1 (7.7)**
• Diaphragm + bone + adenophatic	0 (0.0)	0 (0.0)	1 (7.7)
• Lung + cerebral	0 (0.0)	1 (4.8)	0 (0.0)
• Bone	0 (0.0)	1 (4.8)	0 (0.0)

Data are expressed as n (%) unless otherwise specified
*HCC h*epatocellular carcinoma, *ICC intrahepatic cholangiocarcinoma*

Figures

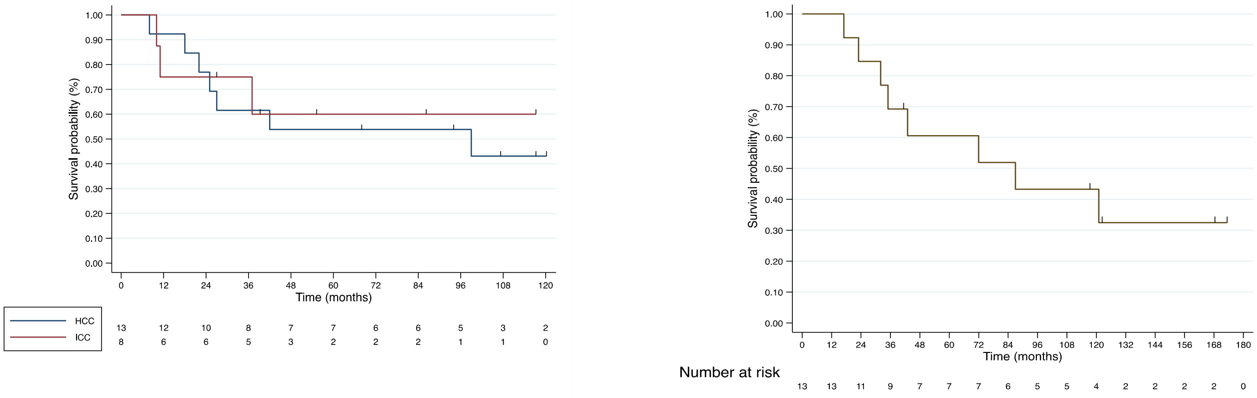

Figure S1. a Kaplan-Meier DFS curves from diagnosis for LR group according to diagnosis. **b** Kaplan-Meier DFS curves from liver transplantation
